# Parallel Evolution of the Genetic Code in Arthropod Mitochondrial Genomes

**DOI:** 10.1371/journal.pbio.0040127

**Published:** 2006-04-25

**Authors:** Federico Abascal, David Posada, Robin D Knight, Rafael Zardoya

**Affiliations:** **1**Departamento de Bioquímica, Genética, e Inmunología, Universidad de Vigo, Vigo, Spain; **2**Departamento de Biodiversidad y Biología Evolutiva, Museo Nacional de Ciencias Naturales, Madrid, Spain; **3**Department of Chemistry and Biochemistry, University of Colorado at Boulder, Boulder, Colorado, United States of America; University of TexasUnited States of America

## Abstract

The genetic code provides the translation table necessary to transform the information contained in DNA into the language of proteins. In this table, a correspondence between each codon and each amino acid is established: tRNA is the main adaptor that links the two. Although the genetic code is nearly universal, several variants of this code have been described in a wide range of nuclear and organellar systems, especially in metazoan mitochondria. These variants are generally found by searching for conserved positions that consistently code for a specific alternative amino acid in a new species. We have devised an accurate computational method to automate these comparisons, and have tested it with 626 metazoan mitochondrial genomes. Our results indicate that several arthropods have a new genetic code and translate the codon AGG as lysine instead of serine (as in the invertebrate mitochondrial genetic code) or arginine (as in the standard genetic code). We have investigated the evolution of the genetic code in the arthropods and found several events of parallel evolution in which the AGG codon was reassigned between serine and lysine. Our analyses also revealed correlated evolution between the arthropod genetic codes and the tRNA-Lys/-Ser, which show specific point mutations at the anticodons. These rather simple mutations, together with a low usage of the AGG codon, might explain the recurrence of the AGG reassignments.

## Abstract 2

The authors find evidence for parallel evolution of an alternate genetic code in arthropod mitochondria (AGG is translated into lysine rather than serine), and correlated co-evolution of the tRNA-Lys/Ser anticodons.

## Introduction

One of the most remarkable properties of the genetic code is that it is the same in the majority of organisms. This remarkable conservation suggests that it was established early in the evolution of life on earth, before the split of the three main domains of life [ 1], and has remained constant since then. This code is not random. Several studies have related the form of the canonical genetic code to stereochemical properties of amino acids and codons, minimization of the impact of mutations, and biosynthetic relationships among the different amino acids (reviewed in [ 2]).

Despite the optimality of the canonical genetic code, several variants exist. These include nuclear variants in certain ciliates and yeasts, and, especially, variants in metazoan mitochondria, where ten different codes have already been identified [ 3]. In animals, mitochondria have compact genomes that typically encode only 13 proteins involved in oxidative respiration [ 4]. The pressure towards genome size reduction in mitochondria, which affects the number of tRNA genes, might explain the high frequency of codon reassignments (change of meaning) in these organelles [ 5]. The small size of mitochondrial genomes might also explain why these reassignments are tolerated rather than deleterious.

However, most codon reassignments in mitochondria are conserved within each metazoan phylum. This conservation has been interpreted to mean that reassignments are rare and that each particular reassignment stems from a single evolutionary event. Here, we demonstrate that the availability of large numbers of complete mitochondrial genomes enables high-resolution studies of the evolution of the genetic code, revealing that codon reassignments may be far more common than previously thought.

Non-standard codes usually arise from changes in the tRNAs [ 6], and some codons seem to be reassigned more frequently than others [ 5]. For instance, the AGA and AGG codons (AGR), which correspond to arginine (Arg) in the standard code, are particularly labile and have been reassigned to serine (Ser), glycine (Gly), and stop codons in different metazoan lineages. Previous work suggested that the change from Arg to Ser occurred only once at the base of the Bilateria, and that the subsequent changes took place within deuterostomes [ 3].

Several mechanisms besides the loss of the ancestral tRNA-Arg [ 7] have been shown to contribute to AGR reassignment in metazoans. Reassignment of AGA to Ser may be a relatively easy change, because one of the serine isoacceptor tRNAs usually has a GCU anticodon. This anticodon, which usually pair with AGC/AGU, can also pair with the AGA codon through noncanonical pairing under certain conditions [ 8]. Reassignment of AGG to Ser has been explained in terms of guanosine methylation of the tRNA-Ser anticodon GCU, which allows it to pair with AGG [ 8, 9]. AGR codons have also been reassigned to Gly in urochordates through the appearance of a new tRNA-Gly with anticodon UCU [ 10]. The use of AGR as stop codons in vertebrate mtDNA may be due to alterations in translation release factors [ 11] or in rRNA [ 12]. In Porifera, AGR codons have the standard Arg meaning because a new tRNA-Arg was recruited from a different tRNA isoacceptor family [ 13], suggesting either that this new tRNA displaced the original tRNA-Arg without change of function or that the original AGR reassignment to Ser occurred even earlier, at the base of Metazoa.

Changes in tRNAs explain how, but not why, most codon reassignments take place. Several hypotheses address this latter question. The codon-capture model [ 14] proposes that mutational biases can eliminate specific codons from the entire genome and then, by neutral evolution, mutations at other tRNA molecules can make them able to recognize such codons, in such a way that when those codons reappear in the genome their meaning has already changed. In contrast, the ambiguous intermediate hypothesis [ 15] suggests that mutations at regions other than the tRNA-anticodon can induce a codon to be ambiguously translated by more than one tRNA, and that later the recognition of that codon by the mutant tRNA can be gradually fixed by natural selection leading to the codon reassignment. Different examples have been cited as support for both hypotheses [ 16, 17].

Genetic code variants are generally found by comparative sequence analysis. When a particular codon occurs at protein sites in which a specific amino acid is consistently found in other related species (e.g., reference [ 18]), the most likely explanation is that this codon has been reassigned, although phenomena such as RNA editing make such inferences hazardous for individual genes. We have automated this comparative process, allowing us to apply it to large numbers of genomes.

## Results

### A Computational Method for Codon Assignment

Our method, described in more detail in the Materials and Methods section, automatically detects variant genetic codes in animals. For each codon in each species, we test which amino acid is most frequently found at homologous positions in other species. This most frequent amino acid is then predicted to be the translation of that codon. Because poorly conserved regions of proteins may introduce some noise in the prediction, we use an entropy-based threshold to eliminate variable columns from the alignments (see Materials and Methods section). We applied this method to each of 626 animal mitochondrial genomes available at NCBI ( http://www.ncbi.nlm.nih.gov) in order to automatically assign their genetic codes.

### Accuracy of the Computational Prediction of the Genetic Code

**Figure 1:**
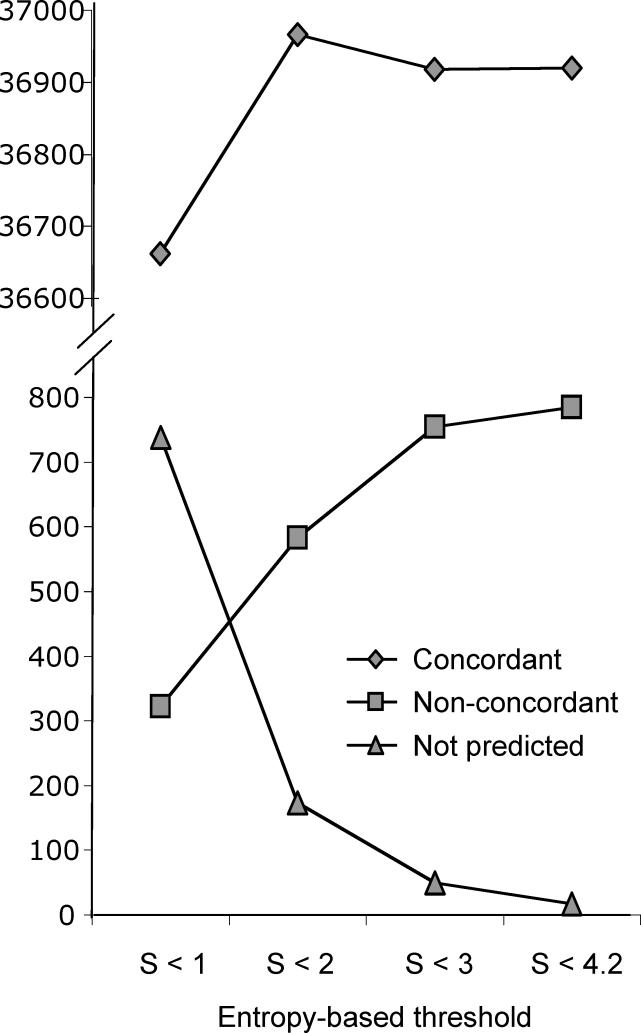
Performance of the Codon Assignment Method The number of assignments concordant (diamonds) and non-concordant (squares) with GenBank annotations, as well as the number of codons left unpredicted (because there were no observations for them; triangles) at different thresholds of entropy are shown.

In order to test the validity of our approach, we compared the automatic assignments with the annotated genetic codes provided in GenBank for each species. Assuming that predictions that are not concordant with GenBank are erroneous (an assumption that is usually, but not always, correct) we were able to estimate the accuracy of the method. In Figure 1 we plot the number of concordant/non-concordant predictions as well as the number of unpredicted codons (those which were not observed at positions below the entropy threshold) for four different entropy-thresholds *(S)*. Overall, most codon assignments were concordant with the annotations in GenBank (e.g., 98.4% for *S* < 2), indicating that the method is highly accurate. This figure also illustrates how under more permissive thresholds (e.g., *S* < 4.32) the number of unpredicted codons decreases. This decrease occurs because fewer alignment columns are discarded and more codon observations become available, and hence the method is able to assign less abundant codons. On the other hand, because columns with higher variability are used to infer the meaning of a codon under more permissive thresholds, the rate of incorrect assignments increases. Similarly, removing gap-rich columns results in a small increase in concordant predictions, despite a slight decrease in the total number of predictions (unpublished data). Excluding columns with more than 20% gaps, thresholds between 1 and 2 represented the most appropriate balance between specificity and sensitivity. For further analyses, we selected the threshold *S* = 2 because it provided the highest number of correct assignments (36.966), and an acceptable number of not predicted codons (173 codons, 0.004%).

**Figure 2:**
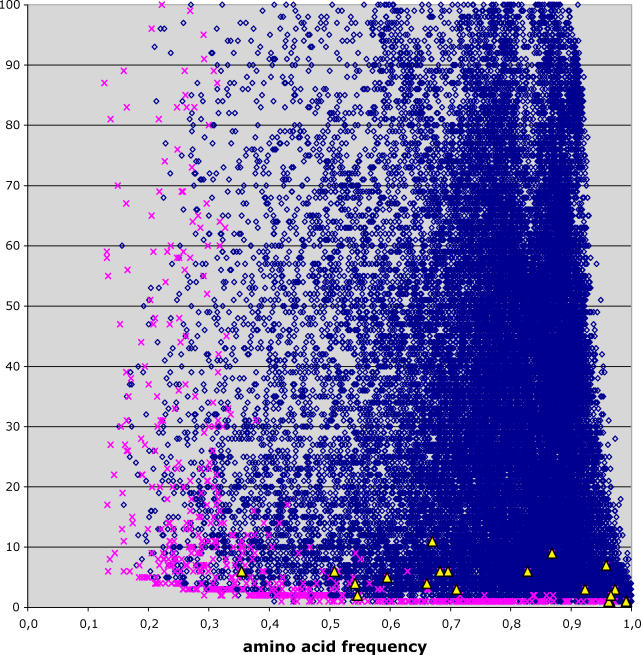
Codon-Usage and Strength of Prediction The number of codons used for each codon assignment and the frequency of the predicted amino acid are shown. Blue diamonds indicate assignments that are concordant with GenBank annotations; pink crosses, discordant assignments; and yellow triangles, discordant assignments where AGG is assigned to lysine.

In order to characterize those assignments that were not concordant with GenBank annotations, we plotted the number of observations (number of codons) against the strength of the signal (amino acid frequency) supporting each codon assignment ( Figure 2). Concordant assignments (blue diamonds) were usually predicted based on a large number of codon observations and/or alignment columns in which the most frequent amino acid was especially common, although the variability was substantial. In contrast, most non-concordant assignments (pink crosses) corresponded to predictions based on either low numbers of codon observations or low amino acid frequencies, i.e. they are unreliable predictions. We noticed that most of the non-concordant predictions occurred in platyhelminths and nematodes ( Table 1). This taxonomic bias could be explained by the extreme divergence between these species and the rest of the metazoa, which has the effect of reducing the number of conserved sites between them. In fact, restricting the analysis to include only platyhelminth and nematode species in the comparisons considerably reduced the number of non-concordant predictions in these phyla (unpublished data).

**Table 1:**
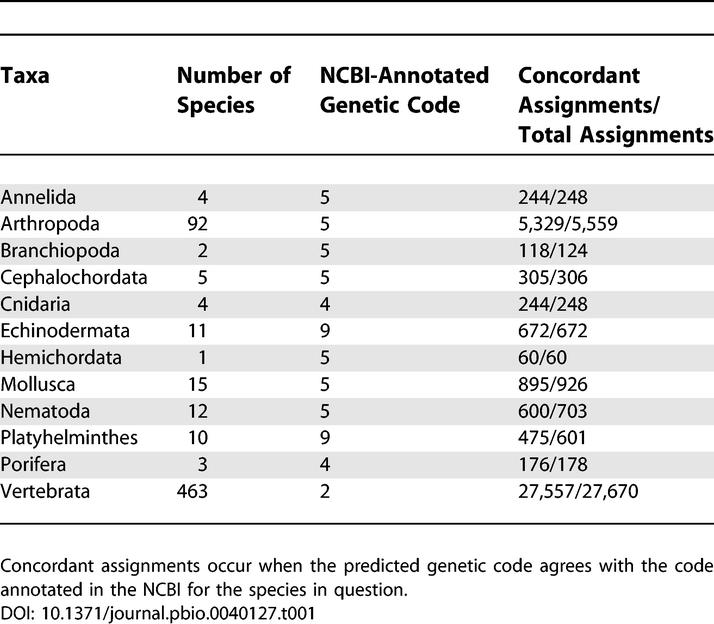
Performance of Codon Assignment Methodology

### A New Genetic Code in Arthropods

Remarkably, some non-concordant assignments occurred at intermediate numbers of codon observations and high amino acid frequency (some of the yellow triangles in Figure 2), in a region where assignments were nearly all correct. All these observations corresponded to changes in the AGG codon. AGG, which translates to Ser according to the Invertebrate Mitochondrial Genetic Code (IMGC), was predicted to translate as Lys in several arthropods in the high-confidence region. Other arthropods were also predicted to have the same change, but were located in a region of Figure 2 in which both correct and incorrect assignments are frequent (the remaining yellow triangles). This particular codon assignment was consistent whether we used all metazoans ( *n* = 626) or only arthropods ( *n* = 92) for the analysis, eliminating the possibility that arthropod assignments were poorly predicted because of their distance from the rest of the metazoans. These codon assignments were also consistent under different conservation thresholds (unpublished data). AGG predicted to code for Lys was also the only non-concordant assignment occurring repeatedly in different species. These observations strongly suggest that AGG to Lys is a new, previously unobserved codon reassignment.

**Figure 3:**
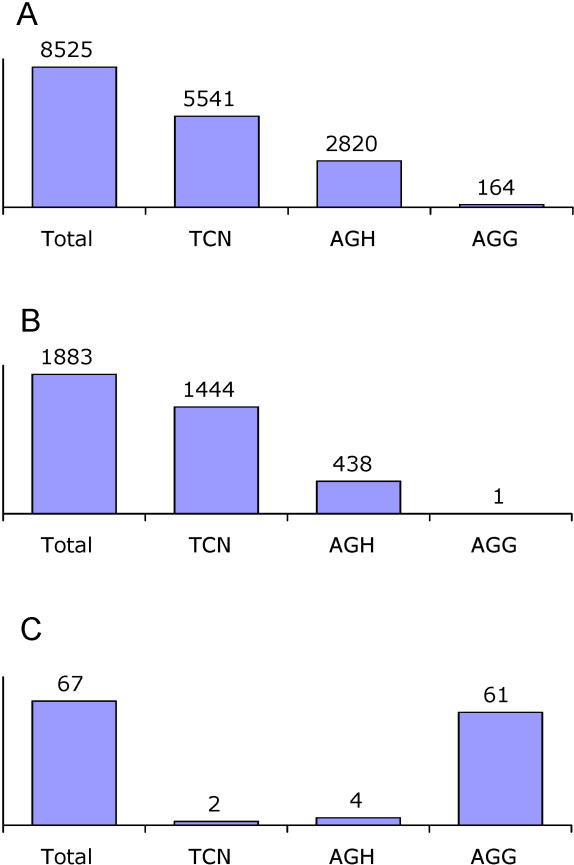
Usage of the TCN and AGN Codons in the 24 Arthropod Mitochondrial Genomes Predicted to Translate AGG as Lys The overall usage of the TCN/AGN codons (A), and their particular usage at protein sites where Ser (B) or Lys (C) are conserved across more than 80% of the 626 analyzed metazoan mtDNAs are shown. N=A, C, G, or T; H=A, C, or T.

According to our results from the arthropod dataset, 24 species translate AGG as Lys, and 34 species translate it as Ser. For 18 species, the meaning of AGG could not be predicted, and 16 species do not use AGG at all. In the species predicted to translate AGG as Lys, TCN and AGH (AGC, AGT, AGA) codons are clearly associated with alignment columns were Ser is > 80% conserved, whereas the AGG codon is distinctly associated with Lys-conserved columns ( Figure 3). The latter effect is exemplified by the crustacean Speleonectes tulumensis. This species has a total of 17 AGG codons, of which nine occur at positions below the entropy threshold. Eight of these nine codons appear at positions where Lys is > 80% conserved. The probability of this happening by chance is very small: there are 37 alignment columns out of a total of 2,443 with more than 80% of Lys, and the probability that after randomly placing nine AGG codons, at least eight of them occupy these Lys-columns is P(X ≥ 8) = 1.5 × 10 ^−17^.

### Evolution of the Genetic Code in Arthropods

**Figure 4:**
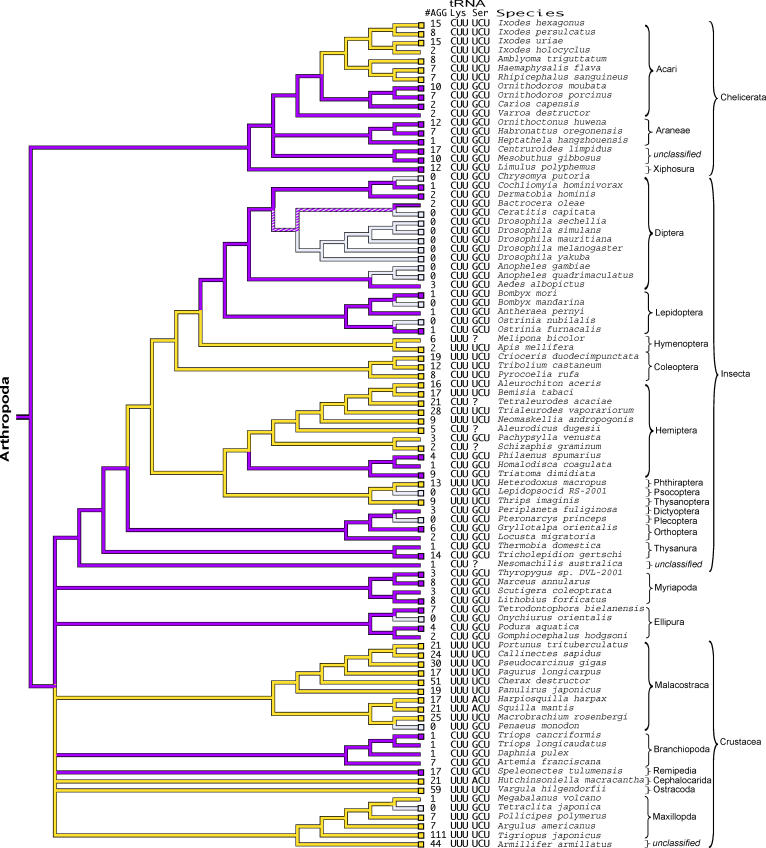
Evolution of the Mitochondrial Genetic Code in Arthropods This figure merges two independent most-parsimonious ancestral character state reconstructions: the presence (transparent) or absence (light grey) of the AGG codon, and the predicted translation of AGG as Lys (purple) or Ser (yellow). Species in which AGG was not predicted and species in which we determined that the assignment was unreliable (nodes without a rectangle) were treated as ambiguous states. The number of AGG codons, and the anticodons of tRNA-Lys and tRNA-Ser are indicated next to the species name.

To further understand the origin and distribution of this new genetic code, we examined its evolution along the arthropod phylogeny. However, the phylogeny of the main lineages of arthropods is controversial. We used a consensus phylogenetic tree that we assembled from different sources [ 19– 26] to best reflect current knowledge of arthropod relationships ( Figure 4). Polytomies were introduced in several cases where uncertainty existed: the relative position of hexapods with respect to myriapods and crustaceans (Atelocerata and Pancrustacean hypotheses, respectively) [ 22], the mono/paraphyly of hexapods (depending on the relative position of Ellipura with respect to insects) [ 20, 21, 27], the monophyly of crustaceans [ 24], and the relationships among the different crustacean classes. The phylogeny of the different orders of insects was mostly based on reference [ 19]. The most parsimonious reconstruction [ 28] of the evolution of the genetic code on this tree clearly indicated that the arthropod mitochondrial genetic code changed multiple times ( Figure 4). Unexpectedly, changes occurred both within the major and minor groups of arthropods. For example, in the order Hemiptera, Euhemiptera *(Philaenus spumarius, Triatoma dimidiata)* read AGG as Lys, but Sternorrhyncha *(Aleurochiton aceris, Bemisia tabaci, Tetraleurodes acaciae, Trialeurodes vaporariorum, Neomaskellia andropogonis, Aleurodicus dugesii, Schizaphis graminum)* read AGG as Ser. A similar pattern was observed in the chelicerate subclass Acari. Interestingly, and regardless of the inclusion of an outgroup species with the IMGC, the ancestral state reconstruction indicated that the genetic code that translates AGG as Lys is the ancestral one.

We confirmed that ancestral state reconstruction gives the same results when resolving polytomies in different ways (according to several recently published arthropod phylogenies [ 20– 22, 24, 29]), and when rooting the tree with an outgroup that has the IMGC. In all but one of the alternative combinations we analyzed, the ancestral state was unambiguously predicted to translate AGG as Lys. The only case in which ancestral reconstruction was ambiguous occurred when neither Remipedia nor Branchiopoda crustacean classes were placed basal to the other crustaceans, and, simultaneously, Chelicerata and Myriapoda were recovered as a monophyletic group. In no case was the IMGC predicted to be the ancestral code of arthropods. We therefore decided to name this novel genetic code the Ancestral Arthropod Mitochondrial Genetic Code (AAMGC) to differentiate it from the IMGC in which AGG is translated as Ser.

The ancestral nature of this AAMGC is further supported by the observation that it is found in many arthropod lineages thought to be early diverging. For example, the most widely accepted basal lineages of Chelicerata (horseshoe crabs -Merostomata-) [ 23], Hexapoda (Ellipura) [ 19], and Insecta (Thysanura) [ 19], are all predicted to use the AAMGC. This is also the case for the classes Remipedia and Branchiopoda, which have been often proposed to be among the most primitive crustaceans [ 30]. Myriapods also use the AAMGC.

### Molecular Basis of the AGG Reassignments

**Figure 5:**
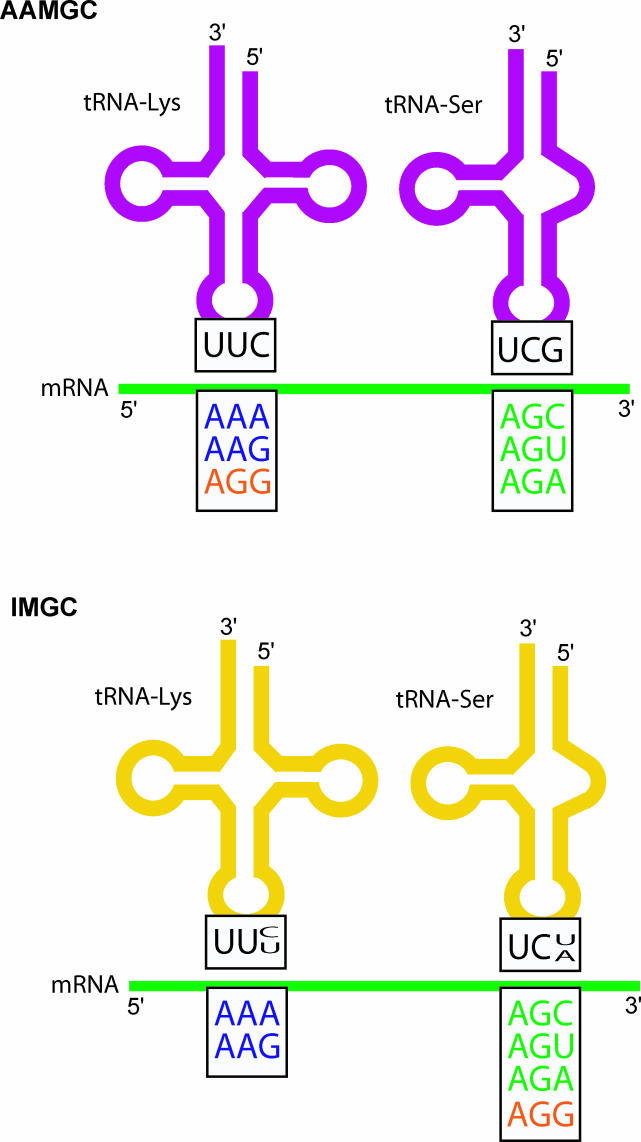
The Molecules of tRNA-Lys and -Ser in Arthropods Having Either the AAMGC or the IMGC The tRNA-Lys and -Ser anticodons in species decoding AGG as Lys or Ser, as well as the predicted translation of AGN and AAR mRNA-codons, are shown. Note that anticodons are depicted in 3′ to 5′ sense, i.e. UUC in tRNA-Lys corresponds to the anticodon CUU in standard notation.

To further understand the molecular basis of the multiple AGG reassignments in arthropods, we analyzed the tRNA-Lys and tRNA-Ser (AGN) sequences ( Figure 4). Using mutual information (see Materials and Methods), we attempted to determine which positions evolved in a concerted manner with the meaning of the AGG codon. We found that mutations at anticodons in both tRNAs were correlated with the evolution of the genetic code. In the case of tRNA-Ser, anticodon mutations were highly diagnostic for AGG codon reassignments (test for independence in the evolution of both characters: *p-*value < 0.005, see Protocol S1 and Figure S1). As summarized in Figure 5, all and only the arthropod species predicted to decode AGG as Ser changed the typical anticodon GCU of the tRNA-Ser (AGN), either to UCU (28 species) or ACU (three species), with only two exceptions. These exceptions were that Pollicipes polymerus was predicted to have the IMGC, but its tRNA-Ser has the anticodon GCU (probably unable to recognize AGG at least without posttranscriptional modifications), and Ixodes holocyclus that contains only two AGG codons that appear at variable positions and could not be assigned by our method, but its tRNA-Ser has the anticodon UCU (expected to recognize AGG). The anticodon of tRNA-Lys was also strongly associated with the meaning of AGG. Most arthropod species (73% of our sample) have a tRNA-Lys with the anticodon CUU, whereas the rest have UUU. All of the species predicted to decode AGG as Lys have the CUU anticodon (likelihood ratio test for independence *p*-value < 0.005, see Protocol S1 and Figure S1), although many species that have the CUU anticodon either do not use AGG or decode it as Ser ( Figure 5).

## Discussion

### Evolutionary History of the Genetic Code in Arthropods

Our results are consistent with the idea that the reassignment of AGG from Ser to Lys occurred at the base of the arthropods, and that it was later reversed several times in different lineages. If this scenario is true, it implies that basal lineages retained the ancestral state consisting of decoding AGG as Lys, whereas some derived lineages recovered the original IMGC.

The observation that divergent lineages repeatedly changed the genetic code back to IMGC instead of maintaining the “new” code (AAMGC) suggests that there might exist some evolutionary advantage for translating AGG as Ser. This change would result in translation of the whole AGN family of codons in the same way, as occurs with many other codon families in the genetic code. However, although Diptera and Lepidoptera are considered derived orders of insects, they either translate AGG as Lys (though at low frequency) or have eliminated it from their genomes. It is possible that these orders retained the ancestral state, but a more parsimonious scenario is that an additional reassignment from Ser to Lys took place in the ancestor of Diptera and Lepidoptera. This observation makes the hypothesis of a selective advantage for translating AGG as Ser less likely, although other factors may override such an advantage. For instance, a low usage of AGG caused by mutational bias towards low GC content would reduce the putative advantages of translating AGG as Ser, providing a greater role for neutral evolution rather than selection.

Interestingly, although AGG codons are generally rare in arthropods mitochondria, the reassigned codons are not always notably rarer than those with the original meaning in sister taxa, as would be predicted by the codon capture model [ 14]. For example, the difference in the number of AGG codons used in the IMGC and AAMGC species in the clades containing *Ixodes* and *Limulus* is not significant (two-tailed t-test, *p*-value = 0.65). This suggests that it is not necessary for the codon AGG to completely disappear in order to be reassigned through point mutation in the anticodon, although the low usage of this codon makes reassignments less likely to be deleterious.

An illustrative example of the lability of the AGG codon exists in *Penaeus monodon,* which lacks the AGG codon, whereas the other Malacostraca species, which are predicted to decode AGG as Ser, use it frequently. Interestingly, P. monodon has a different tRNA-Ser than the other Malacostraca and has a tRNA-Lys that is probably unable to decode AGG. These changes in tRNA complement suggest that natural selection, rather than directional mutation pressure, contributes to the codon's rarity in this species. The recently sequenced mt-genome of *Marsupenaeus japonicus,* also from the *Penaeidae* family, might help understanding such evolutionary mechanisms. This species has the same anticodons as P. monodon at tRNA-Lys/-Ser. However, three instances of the AGG codon appear in its mt-genome, which are predicted to be translated as Ser. These observations suggest that M. japonicus is at an intermediate stage before the complete elimination of AGG from its genome, and that its tRNA-Ser with the anticodon GCU might retain some ability to recognize the AGG codon.

### Role of tRNAs in the Reassignments of AGG

The molecular basis of AGG reassignment seems to be anticodon mutation rather than the alternative mechanisms of anticodon base modification previously described in starfish [ 8] and squid [ 9], or tRNA mutations leading to ambiguities in translation as expected by the ambiguous intermediate hypothesis [ 15]. The strong association between mutation at the tRNA-Ser anticodon and translation of AGG suggest that this rather simple molecular change alone could explain the recurrence of the reassignment of AGG in arthropods from Lys to Ser. This mechanism requires that the mutated tRNA-Ser (AGN) has more affinity for AGG than the tRNA-Lys, because tRNA-Lys might be still able to recognize the AGG codon. Indeed, the affinity of tRNA-Lys for AGG must be low, since a wobble pairing GU is required at the middle position of the codon. Hence, the tRNA-Ser might be seen as the dominant tRNA.

We also found that some species have the pre-required tRNA-Lys with anticodon CUU and cannot translate AGG as Ser, but nonetheless do not use AGG at all. For instance, we found that the absence or low-usage of AGG in Diptera and Lepidoptera is not exclusively related to low GC content (see Protocol S2 and Table S1). The few flies and butterflies using AGG are all predicted to decode it as Lys, but they use AGG at very low frequency. This observation might be interpreted in two ways. First, the CUU anticodon might be necessary but not sufficient to allow tRNA-Lys to recognize AGG. Changes at other regions may also influence the affinity of the tRNA for its codons [ 31] although neither the primary nor the secondary structure analyses ( Protocol S3, Figures S2 and S3) revealed further correlations. Second, the efficiency of tRNA-Lys in recognizing AGG may always be poor, and hence in some species the elimination of AGG from the genome is preferred. In fact, the codon-anticodon pairing between CUU and AGG is unfavorable and, as far as we know, such a wobble pairing at the middle position of a codon would be unprecedented. It is possible that subtle structural changes throughout the rest of the tRNA body are required to allow the flexibility required for a GU pair at the second position, that a posttranscriptional modification to the tRNA (such as C to U deamination) occurs, or that the mitochondrial-encoded tRNA is non-functional and that a nuclear-encoded tRNA is imported into the mitochondrion and used for translation instead.

### Conclusions

Many arthropods, including those that use the new genetic code, make limited use of AGG. This low abundance makes its assignment particularly difficult from a statistical point of view, and explains why previous characterizations of the genetic code in these species were unable to uncover the AAMGC. Only a global approach such as the one conducted here, which benefits from the comparison of assignments across multiple species, could determine the translation of AGG with high confidence.

Before this study, every known genetic code change in metazoan mitochondria was conserved within a phylum. We show that genetic code variants can be also found among lineages within a metazoan phylum. The main importance of this finding is thus that genetic code changes may be much more frequent than previously suspected, and that the large number of whole-genome sequences that are now available make this kind of high-resolution mapping of genetic code changes possible for the first time. It is perhaps not unexpected that most of the new code changes are found in arthropods, which are numerous and diverse. However, the number of genetic code changes within this clade (even including changes within a single insect order) provide an unprecedented example of parallel mitochondrial genetic code changes within a phylum, and suggest that similar phenomena will be found as more sequences become available in other lineages. Similar patterns of repeated reassignment have previously been observed in the nucleus, in ciliates [ 32, 33] and yeast [ 17], although in ciliates the recurrent changes involved reassignments between sense and stop codons, which have been related to changes in release factors [ 32]. The findings presented here represent an extraordinary case of multiple sense-to-sense reassignments. Interestingly, arthropod species with the AAMGC may use a codon-anticodon interaction never observed before in which a wobble pairing occurs at the middle position of the codon. For all these reasons, the mitochondrial genetic code of arthropods represents a paradigm for further insights into the evolution of the genetic code.

## Materials and Methods

### Data

A total of 626 metazoan mitochondrial genomes were retrieved from NCBI ( http://www.ncbi.nlm.nih.gov) and parsed with the program Mitobank (available at http://darwin.uvigo.es) built using the BioPerl library [ 34]. Multiple alignments for each protein-coding gene were produced with ClustalW [ 35]. We constructed four different sequence datasets comprising metazoans ( *n* = 626), platyhelminths ( *n* = 10), nematodes ( *n* = 12) and arthropods ( *n* = 92).

### Prediction of the mitochondrial genetic code

For a given species of unknown genetic code, our assignment method locates each of the 64 codons in the multiple alignments of homologous proteins. Next, the method calculates the overall frequency of every amino acid in all the columns where a given codon occurs. The amino acid most frequently occurring in related species is then assumed to be the most likely meaning of the codon. Columns of the alignment with more than 20% gaps or with Shannon entropy *(S)* higher than 2 were interpreted as highly variable or poorly aligned, and excluded from further analyses (see Results for a justification). The meaning of a codon is defined as “not predicted” if the codon is used in a given species but not at positions below the entropy-based threshold.

### Phylogenetic reconstruction of ancestral character states

The program Mesquite v1.05 [ 36] was used to build a composite arthropod phylogeny, and to assign optimal character states to the internal nodes of the trees using most-parsimonious reconstructions [ 28] under the Fitch parsimony criterion [ 37].

### Molecular analysis of tRNA-Lys/-Ser

The evolution of tRNA-Lys and -Ser sequences was analysed in order to find positions that evolved concertedly with the genetic code. The predicted translation of AGG was included as a new character in the multiple sequence alignments of tRNA-Lys and -Ser and the mutual information of this character versus the others was calculated with the program MatrixPlot [ 38]. Different criteria for mutual information calculation resulted in similar results.

### Test for the correlated evolution of characters

Rather than counting each species as an independent observation (as in the MatrixPlot analyses), we tested in a phylogenetic context the correlated evolution of those positions identified with MatrixPlot. In order to be able to use the program Discrete [ 39], we built a phylogeny of arthropods in which polytomies were resolved and ambiguities in the studied characters (genetic code or tRNA-anticodon assignment) eliminated ( Figure S1). Branch lengths of this tree were optimized by maximum likelihood with the program Phyml [ 40]. The program Discrete was applied to test for the independence of evolution of genetic code and tRNA-Lys/-Ser anticodons in a maximum likelihood context (see Protocol S1).

## Supporting Information

Figure S1 Phylogenetic Tree Used to Test for Correlated Evolution Polytomies from the tree of Figure 4 were resolved as shown in this figure. The length of the branches was estimated by maximum likelihood. The short length of the internal branches linking different groups of species highlights the difficulties in determining the phylogeny of arthropods.
(125 KB PDF)

Figure S2 Secondary Structure of tRNA-Lys The arthropod tRNA-Lys multiple alignment highlighting the main secondary structure elements of tRNA as well as the predicted translation of the AGG codon is shown.
(3 MB PDF)

Figure S3 Secondary Structure of tRNA-Ser The arthropod tRNA-Ser multiple alignment highlighting the main secondary structure elements of tRNA as well as the predicted translation of the AGG codon is shown.
(3 MB PDF)

Protocol S1 Correlated Evolution of the Genetic Code and the tRNA-Lys/-Ser Anticodons (49 KB DOC)

Protocol S2 GC Content and Absence of AGG in *Diptera* and *Lepidoptera* Orders
(28 KB DOC)

Protocol S3 Secondary Structure of tRNA-Lys/-Ser (26 KB DOC)

Table S1 AGG and TCG Usage in Species with GC-Content at Third Position of Codon Lower than 10% (47 KB DOC)

## Abbreviations

AAMGC: ancestral arthropod mitochondrial genetic code
IMGC: Invertebrate Mitochondrial Genetic Code
