# Supplementary material for: Parallel Evolution of the Genetic Code in Arthropod Mitochondrial Genomes
Source: PLoS Biol. 2006 Apr 25;4(5):e127. doi: 10.1371/journal.pbio.0040127 (PMC1440934; doi:10.1371/journal.pbio.0040127)
Supplement: Supplementary file 1 [file pbio.0040127.sg001.pdf]

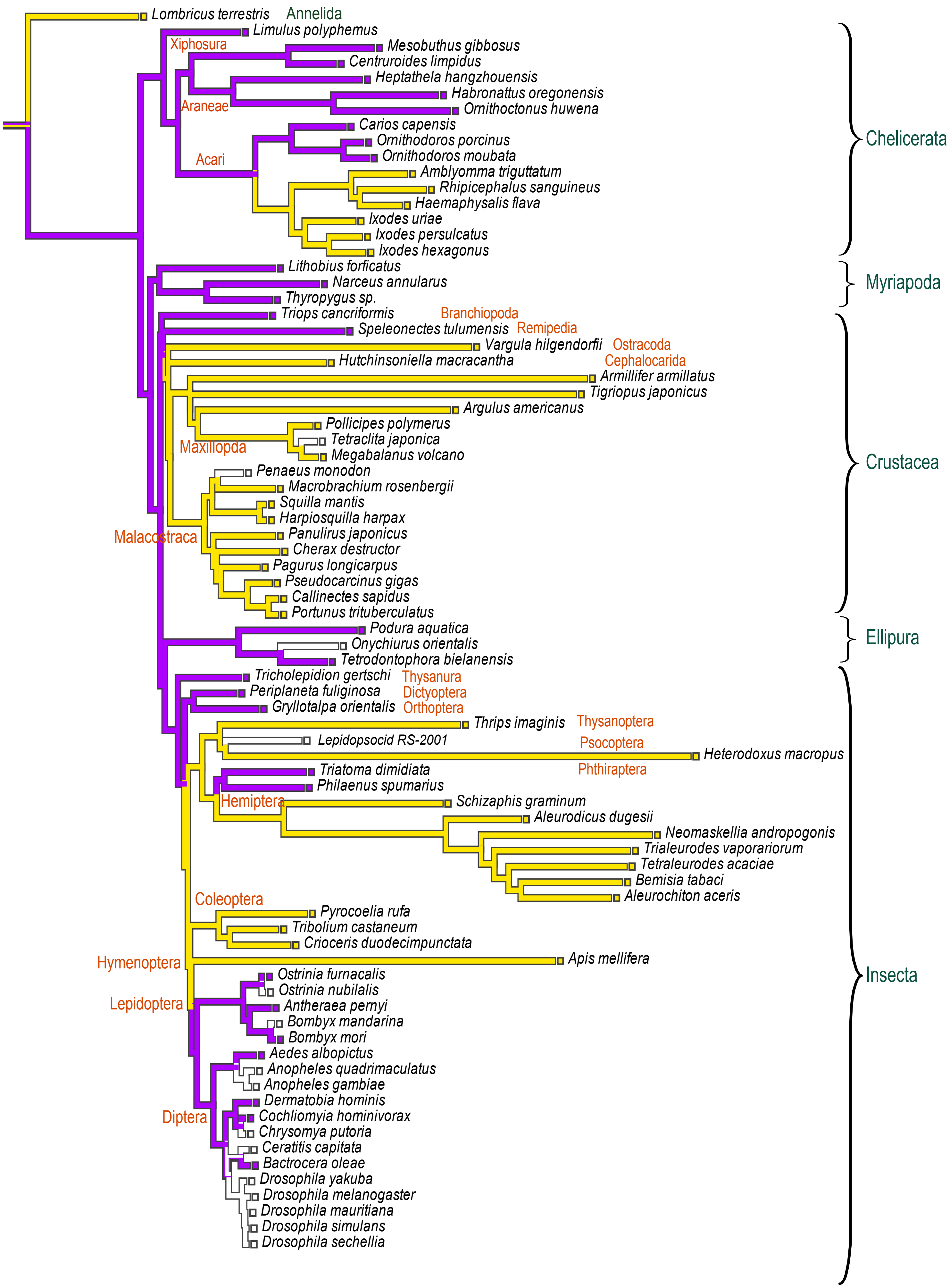
Lombricus terrestris
Annelida
Limulus polyphemus
Xiphosura
Mesobuthus gibbosus
Centruroides limpidus
Heptathela hangzhouensis
Habronattus oregonensis
Ornithoctonus huwena
Araneae
Carios capensis
Ornithodoros porcinus
Ornithodoros moubata
Acari
Amblyomma triguttatum
Rhipicephalus sanguineus
Haemaphysalis flava
Ixodes uriae
Ixodes persulcatus
Ixodes hexagonus
Chelicerata
Lithobius forficatus
Narceus annularus
Thyropygus sp.
Myriapoda
Triops cancriformis
Branchiopoda
Speleonectes tulumensis
Remipedia
Vargula hilgendorfii
Ostracoda
Hutchinsoniella macracantha
Cephalocarida
Armillifer armillatus
Tigriopus japonicus
Argulus americanus
Pollicipes polymerus
Tetraclita japonica
Megabalanus volcano
Maxillopda
Penaeus monodon
Macrobrachium rosenbergii
Squilla mantis
Harpiosquilla harpax
Malacostraca
Panulirus japonicus
Cherax destructor
Pagurus longicarpus
Pseudocarcinus gigas
Callinectes sapidus
Portunus trituberculatus
Crustacea
Podura aquatica
Onychiurus orientalis
Tetrodontophora bielanensis
Ellipura
Tricholepidion gertschi
Thysanura
Periplaneta fuliginosa
Dictyoptera
Gryllotalpa orientalis
Orthoptera
Thrips imaginis
Thysanoptera
Lepidopsocid RS-2001
Psocoptera
Heterodoxus macropus
Phthiraptera
Triatoma dimidiata
Philaenus spumarius
Hemiptera
Schizaphis graminum
Aleurodicus dugesii
Neomaskellia andropogonis
Trialeurodes vaporariorum
Tetraleurodes acaciae
Bemisia tabaci
Aleurochiton aceris
Coleoptera
Pyrocoelia rufa
Tribolium castaneum
Crioceris duodecimpunctata
Hymenoptera
Apis mellifera
Ostrinia furnacalis
Ostrinia nubilalis
Lepidoptera
Antheraea pernyi
Bombyx mandarina
Bombyx mori
Aedes albopictus
Anopheles quadrimaculatus
Anopheles gambiae
Dermatobia hominis
Diptera
Cochliomyia hominivorax
Chrysomya putoria
Ceratitis capitata
Bactrocera oleae
Drosophila yakuba
Drosophila melanogaster
Drosophila mauritiana
Drosophila simulans
Drosophila sechellia
Insecta
