# Supplementary material for: Parallel Evolution of the Genetic Code in Arthropod Mitochondrial Genomes
Source: PLoS Biol. 2006 Apr 25;4(5):e127. doi: 10.1371/journal.pbio.0040127 (PMC1440934; doi:10.1371/journal.pbio.0040127)
Supplement: Supplementary file 2 [file pbio.0040127.sg002.pdf]

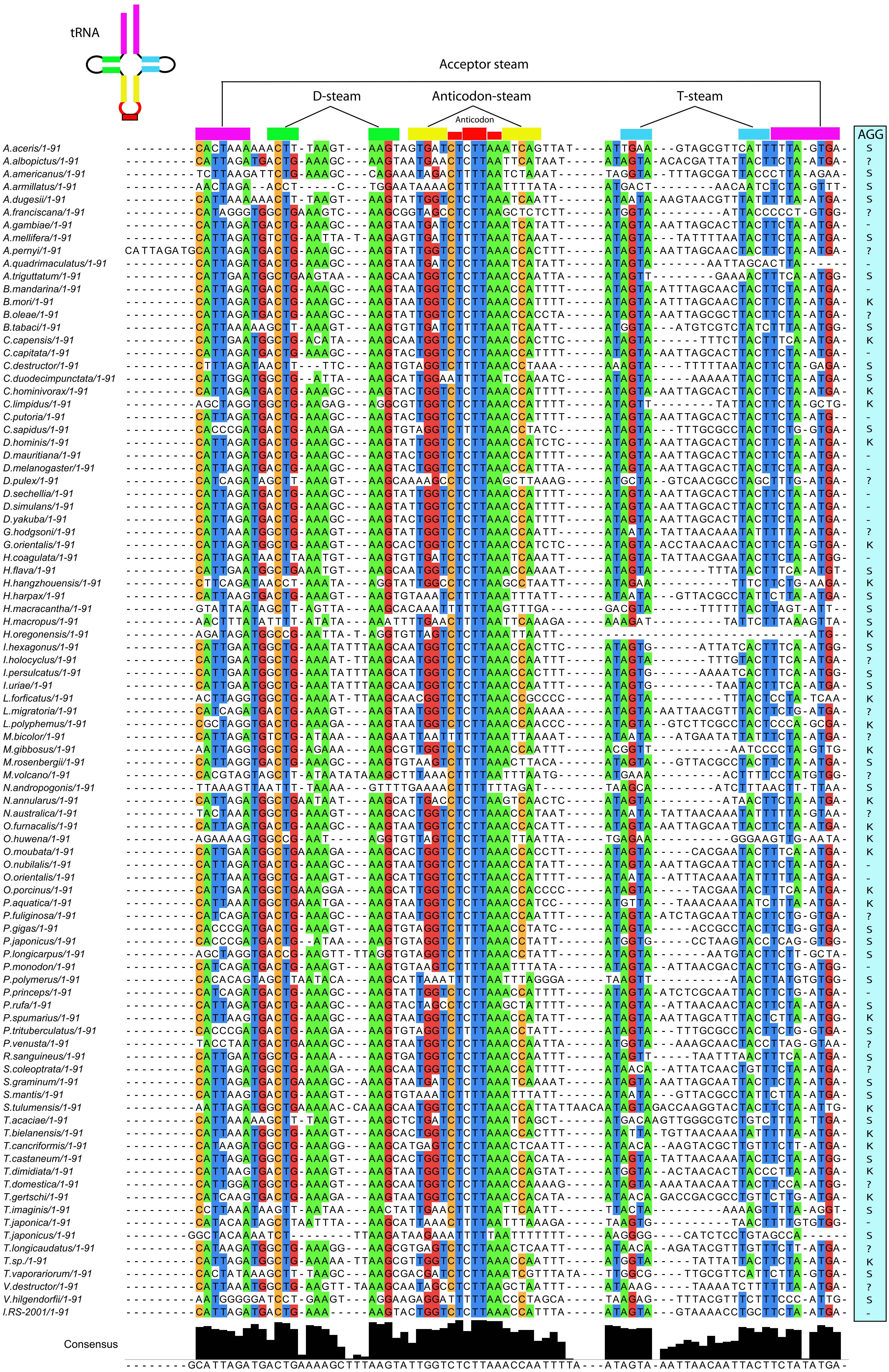
tRNA
Acceptor steam
D-steam
Anticodon-steam
Anticodon
T-steam
AGG
A.aceris/1-91
A.albopictus/1-91
A.americanus/1-91
A.armillatus/1-91
A.dugesii/1-91
A.franciscana/1-91
A.gambiae/1-91
A.mellifera/1-91
A.pernyi/1-91
A.quadrimaculatus/1-91
A.triguttatum/1-91
B.mandarina/1-91
B.mori/1-91
B.oleae/1-91
B.tabaci/1-91
C.capensis/1-91
C.capitata/1-91
C.destructor/1-91
C.duodecimpunctata/1-91
C.hominivorax/1-91
C.limpidus/1-91
C.putoria/1-91
C.sapidus/1-91
D.hominis/1-91
D.mauritiana/1-91
D.melanogaster/1-91
D.pulex/1-91
D.sechellia/1-91
D.simulans/1-91
D.yakuba/1-91
G.hodgsoni/1-91
G.orientalis/1-91
H.coagulata/1-91
H.flava/1-91
H.hangzhouensis/1-91
H.harpax/1-91
H.macracantha/1-91
H.macropus/1-91
H.oregonensis/1-91
I.hexagonus/1-91
I.holocyclus/1-91
I.persulcatus/1-91
I.uriae/1-91
L.forficatus/1-91
L.migratoria/1-91
L.polyphemus/1-91
M.bicolor/1-91
M.gibbosus/1-91
M.rosenbergii/1-91
M.volcano/1-91
N.andropogonis/1-91
N.annulurus/1-91
N.australica/1-91
O.furnacalis/1-91
O.huwena/1-91
O.moubata/1-91
O.nubilalis/1-91
O.orientalis/1-91
O.porcinus/1-91
P.aquatica/1-91
P.fuliginosa/1-91
P.gigas/1-91
P.japonicus/1-91
P.longicarpus/1-91
P.monodon/1-91
P.polymerus/1-91
P.princeps/1-91
P.rufa/1-91
P.spumarius/1-91
P.trituberculatus/1-91
P.venusta/1-91
R.sanguineus/1-91
S.coleoptrata/1-91
S.graminum/1-91
S.mantis/1-91
S.tulumensis/1-91
T.acaciae/1-91
T.bielanensis/1-91
T.cancriformis/1-91
T.castaneum/1-91
T.dimidiata/1-91
T.domestica/1-91
T.gertschi/1-91
T.imaginis/1-91
T.japonica/1-91
T.japonicus/1-91
T.longicaudatus/1-91
T.sp./1-91
T.vaporariorum/1-91
V.destructor/1-91
V.hilgendorfii/1-91
I.RS-2001/1-91
Consensus
--------GCATTAGATGACTGAAAAGCTTTAAGTATTGGTCTCTTAAACCAATTTTA---ATAGTA-AATTAACAATTACTTCTATATGA-
