# Supplementary material for: Parallel Evolution of the Genetic Code in Arthropod Mitochondrial Genomes
Source: PLoS Biol. 2006 Apr 25;4(5):e127. doi: 10.1371/journal.pbio.0040127 (PMC1440934; doi:10.1371/journal.pbio.0040127)
Supplement: Supplementary file 3 [file pbio.0040127.sg003.pdf]

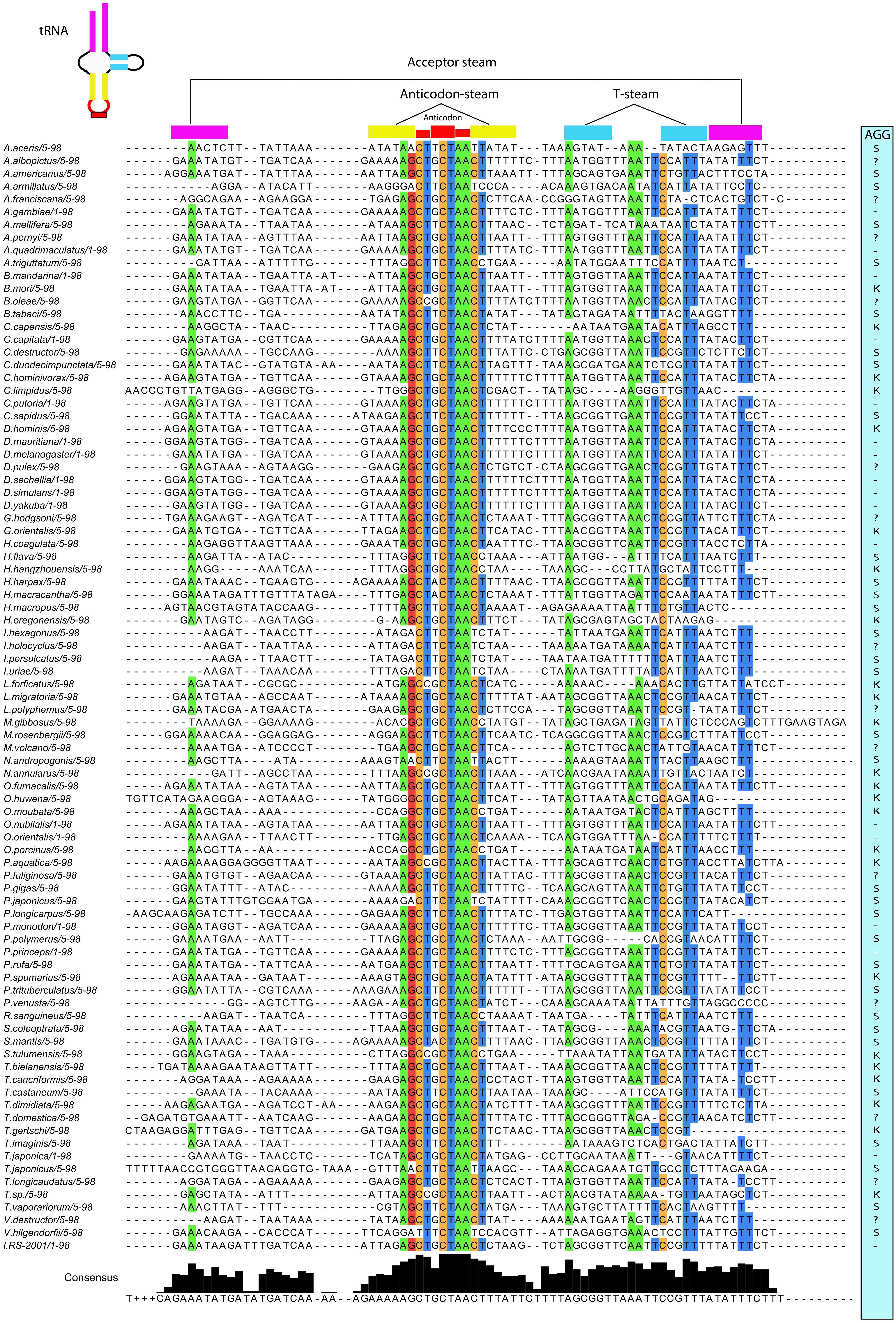
tRNA
Acceptor steam
Anticodon-steam
Anticodon
T-steam
AGG
A.aceris/5-98
A.albopictus/5-98
A.americanus/5-98
A.armillatus/5-98
A.franciscana/5-98
A.gambiae/1-98
A.mellifera/5-98
A.pernyi/5-98
A.quadrimaculatus/1-98
A.triguttatum/5-98
B.mandarina/1-98
B.mori/5-98
B.oleae/5-98
B.tabaci/5-98
C.capensis/5-98
C.capitata/1-98
C.destructor/5-98
C.duodecimpunctata/5-98
C.hominivorax/5-98
C.limpidus/5-98
C.putoria/1-98
C.sapidus/5-98
D.hominis/5-98
D.mauritiana/1-98
D.melanogaster/1-98
D.pulex/5-98
D.sechellia/1-98
D.simulans/1-98
D.yakuba/1-98
G.hodgsoni/5-98
G.orientalis/5-98
H.coagulata/5-98
H.flava/5-98
H.hangzhouensis/5-98
H.harpax/5-98
H.macracantha/5-98
H.macropus/5-98
H.oregonensis/5-98
I.hexagonus/5-98
I.holocyclus/5-98
I.persulcatus/5-98
I.uriae/5-98
L.forficatus/5-98
L.migratoria/5-98
L.polyphemus/5-98
M.gibbosus/5-98
M.rosenbergii/5-98
M.volcano/5-98
N.andropogonis/5-98
N.annularus/5-98
O.furnacalis/5-98
O.huwena/5-98
O.moubata/5-98
O.nubilalis/1-98
O.orientalis/1-98
O.porcinus/5-98
P.aquatica/5-98
P.fuliginosa/5-98
P.gigas/5-98
P.japonicus/5-98
P.longicarpus/5-98
P.monodon/1-98
P.polymerus/5-98
P.princeps/1-98
P.rufa/5-98
P.spumarius/5-98
P.trituberculatus/5-98
P.venusta/5-98
R.sanguineus/5-98
S.coleoptrata/5-98
S.mantis/5-98
S.tulumensis/5-98
T.bielanensis/5-98
T.cancriformis/5-98
T.castaneum/5-98
T.dimidiata/5-98
T.domestica/5-98
T.gertschi/5-98
T.imaginis/5-98
T.japonica/1-98
T.japonicus/5-98
T.longicaudatus/5-98
T.sp./5-98
T.vaporariorum/5-98
V.destructor/5-98
V.hilgendorfii/5-98
I.RS-2001/1-98
Consensus
T+++CAGAAATATGATATGATCAA-AA--AGAAAAAGCTGCTAACTTTATTCTTTTAGCGGTTAAATTCCGTTTATATTTCTTT---------
