# Supplementary material for: Parallel Evolution of the Genetic Code in Arthropod Mitochondrial Genomes
Source: PLoS Biol. 2006 Apr 25;4(5):e127. doi: 10.1371/journal.pbio.0040127 (PMC1440934; doi:10.1371/journal.pbio.0040127)
Supplement: Supplementary file 4 [file pbio.0040127.sd001.doc]

**Supplementary Protocol S1. Correlated evolution of genetic code and tRNA-Lys/-Ser anticodons.**

We tested for correlations between the evolution of the genetic code and tRNA-Lys and Ser in a phylogenetic context, rather than just counting each species as independent observations. Specifically, we performed a likelihood ratio test between two models of character evolution. In the null model the tRNA (-Lys or -Ser) anticodon has evolved independently of the genetic code, while in the alternative model the evolution of the genetic code and the tRNA anticodon are correlated. These calculations are implemented in the program Discrete [1]. In order to calculate model likelihoods we removed any source of ambiguity, either in the tree (polytomies) or in the studied characters (genetic code or tRNA-anticodon assignment).

The phylogeny of arthropods was resolved as shown in figure S1, by placing chelicerates as the basal group of arthropods [2]. For simplicity, crustaceans were treated as monophyletic even though they have also been proposed to be paraphyletic (reviewed in [3]). The relationships among the different orders of Crustacea were established in a way that the number of genetic code changes was minimized, what represents a conservative scenario when testing for correlated evolution. Collembolas (Ellipura) were treated as the sister group of insects [4,5]. As an outgroup we included the annelid *Lumbricus terrestris* in which AGG is translated as Ser, tRNA-Lys has anticodon UUU and tRNA-Ser(AGN) anticodon UCU. Importantly, repeating the analysis using different variations of these phylogenies did not affect the outcome of the correlation test (e.g. placing Myriapoda as the basal group, or placing Ellipura as the sister group of Insecta+Crustacea).  To estimate the branch lengths in this tree, we first built a protein alignment of the arthropod mitochondrial genomes using ClustalX [6] and Gblocks [7]. The best-fit model of protein evolution was selected with ProtTest [8-10]. Branch lengths were optimized by maximum likelihood under the best-fit model (MtREV+I+G) in Phyml [9]. Then we pruned from the tree those species for which either the meaning of AGG could not be predicted or we were unable to determine the anticodon of tRNA-Ser. This resulted in two trees of 64 and 61 species for the tRNA-Lys (dataset 1) and tRNA-Ser (dataset 2), respectively.

After likelihood optimization in Discrete, the resulting likelihood ratios (LR) for the correlated evolution of the genetic code with tRNA-Lys and Ser were 9.5 and 17.2, respectively. The omnibus test [1] (based on the observation that twice the LR follows a chi-square distribution with four degrees of freedom) indicates that the resulting probabilities of observing this values under the independent evolution model are less than 0.005. Additionally, after simulating the null distribution of the LR statistic using Monte Carlo (100 replicates), the estimated p-values were less than 0.01 in both cases (i.e., no simulated data resulted in a LR higher than the one obtained for the original datasets). Therefore, the evolution of the genetic code and the evolution of the tRNA-Ser and Lys anticodons, are significantly correlated.

1. Pagel M (1994) Detecting correlated evolution on phylogenies: a general method for the comparative analysis of discrete characters. Proc Biol Sci 255: 37-45.

2. Giribet G, Edgecombe GD, Wheeler WC (2001) Arthropod phylogeny based on eight molecular loci and morphology. Nature 413: 157-161.

3. Martin JW, Davis GE (2001) An updated Classification of the Recent Crustacea. Los Angeles, CA.: Natural History Museum of Los Angeles County.

4. Delsuc F, Phillips MJ, Penny D (2003) Comment on "Hexapod origins: monophyletic or paraphyletic?" Science 301: 1482; author reply 1482.

5. Regier JC, Shultz JW (2001) Elongation factor-2: a useful gene for arthropod phylogenetics. Mol Phylogenet Evol 20: 136-148.

6. Thompson JD, Higgins DG, Gibson TJ (1994) CLUSTAL W: improving the sensitivity of progressive multiple sequence alignment through sequence weighting, position-specific gap penalties and weight matrix choice. Nucleic Acids Res 22: 4673-4680.

7. Castresana J (2000) Selection of conserved blocks from multiple alignments for their use in phylogenetic analysis. Mol Biol Evol 17: 540-552.

8. Abascal F, Zardoya R, Posada D (2005) ProtTest: selection of best-fit models of protein evolution. Bioinformatics 21: 2104-2105.

9. Guindon S, Gascuel O (2003) A simple, fast, and accurate algorithm to estimate large phylogenies by maximum likelihood. Syst Biol 52: 696-704.

10. Drummond A, Strimmer K (2001) PAL: an object-oriented programming library for molecular evolution and phylogenetics. Bioinformatics 17: 662-663.
