# Supplementary material for: Parallel Evolution of the Genetic Code in Arthropod Mitochondrial Genomes
Source: PLoS Biol. 2006 Apr 25;4(5):e127. doi: 10.1371/journal.pbio.0040127 (PMC1440934; doi:10.1371/journal.pbio.0040127)
Supplement: Supplementary file 5 [file pbio.0040127.sd002.doc]

**Supplementary Protocol 2. GC content and absence of AGG in *Diptera* and *Lepidoptera* orders**

Codon usage in mtDNA is clearly correlated with GC content. To determine whether the absence of AGG in the majority of flies and butterflies is related to low GC-content, we compared the AGG usage in species having similar GC content (at the third codon position). The usage of TCG (which has the same GC content) was used for comparison. In *Diptera* and *Lepidoptera* the codon AGG is clearly less used than TCG (two-tailed t-test P-value < 0.001). However, in the other species, AGG appears at least as frequently as TCG (two-tailed t-test P-value = 0.12). Therefore, these results suggest that the low usage of AGG in *Diptera* and *Lepidoptera* is not solely dependent on GC content.
