# Supplementary material for: Parallel Evolution of the Genetic Code in Arthropod Mitochondrial Genomes
Source: PLoS Biol. 2006 Apr 25;4(5):e127. doi: 10.1371/journal.pbio.0040127 (PMC1440934; doi:10.1371/journal.pbio.0040127)
Supplement: Supplementary file 6 [file pbio.0040127.sd003.doc]

**Supplementary Protocol S3. Secondary structure of tRNA-Lys/-Ser**

Although the primary structure analysis of tRNA revealed that mutations at anticodon regions alone can explain the evolution of the genetic code, we also analysed the secondary structure of tRNA-Lys (Figure S2) and tRNA-Ser (Figure S3) for potential subtle correlations. We mapped onto the multiple alignments of both tRNAs the location of the different secondary structure elements (acceptor, D-arm, T-arm and anticodon stems), which where identified visually and with the help of Pfold [1], as well as the predicted translation of the AGG codon. Note that even though alignments were corrected by hand, some regions still remain ambiguously aligned. Alignments were visualized and edited with Jalview version 2.07 [2].

We did not find any correlation between the secondary structure of tRNA-Lys/-Ser and the translation of AGG. Some species have their own idiosyncratic structures, but these deviations are not associated with particular genetic codes. For instance, in the case of tRNA-Lys, we found that *H. oregonensis* lack the T-arm as well as the 3’-acceptor stem [3]. Similarly, *O. huwena*, also within the order of *Aranae*, lacks the T-arm. Both species translate AGG as Lys, but the absence of the T-arm is not a characteristic shared by other species that have the same genetic code.

1. Knudsen B, Hein J (2003) Pfold: RNA secondary structure prediction using stochastic context-free grammars. Nucleic Acids Res 31: 3423-3428.

2. Clamp M, Cuff J, Searle SM, Barton GJ (2004) The Jalview Java alignment editor. Bioinformatics 20: 426-427.

3. Masta SE, Boore JL (2004) The complete mitochondrial genome sequence of the spider Habronattus oregonensis reveals rearranged and extremely truncated tRNAs. Mol Biol Evol 21: 893-902.
