# Supplementary material for: Parallel Evolution of the Genetic Code in Arthropod Mitochondrial Genomes
Source: PLoS Biol. 2006 Apr 25;4(5):e127. doi: 10.1371/journal.pbio.0040127 (PMC1440934; doi:10.1371/journal.pbio.0040127)
Supplement: Supplementary file 7 [file pbio.0040127.st001.doc]

| Supplementary Table 1. AGG and TCG usage in species with GC-content at third position of codon lower than 10%. | | | | |
| --- | --- | --- | --- | --- |
| Species | AGG | TCG | GC3 (%) | Order |
| *V.destructor* | 2 | 4 | 9 | *Acari* |
| *B.mandarina* | 0 | 2 | 7 | *Lepidoptera* |
| *O.nubilalis* | 0 | 5 | 7 | *Lepidoptera* |
| *B.mori* | 1 | 1 | 7 | *Lepidoptera* |
| *O.furnacalis* | 1 | 4 | 7 | *Lepidoptera* |
| *A.pernyi* | 1 | 2 | 8 | *Lepidoptera* |
| *D.simulans* | 0 | 0 | 5 | *Diptera* |
| *D.mauritiana* | 0 | 4 | 6 | *Diptera* |
| *D.melanogaster* | 0 | 5 | 6 | *Diptera* |
| *D.yakuba* | 0 | 3 | 6 | *Diptera* |
| *A.albopictus* | 3 | 3 | 6 | *Diptera* |
| *A.gambiae* | 0 | 5 | 7 | *Diptera* |
| *C.capitata* | 0 | 2 | 7 | *Diptera* |
| *D.sechellia* | 0 | 3 | 7 | *Diptera* |
| *C.putoria* | 0 | 3 | 9 | *Diptera* |
| *A.dugesii* | 5 | 2 | 5 | *Hemiptera* |
| *S.graminum* | 2 | 2 | 6 | *Hemiptera* |
| *N.andropogonis* | 9 | 3 | 9 | *Hemiptera* |
| *M.bicolor* | 6 | 0 | 3 | *Hymenoptera* |
| *A.mellifera* | 2 | 1 | 5 | *Hymenoptera* |
| *A.americanus* | 7 | 4 | 9 | *Maxillopoda* |
| *H.macropus* | 13 | 7 | 9 | *Phthiraptera* |
| *L.RS-2001* | 0 | 4 | 7 | *Psocoptera* |
| **Mean for all species** | **2.26** | **3** | **6.83** |  |
| **Mean in Diptera+Lepidoptera** | **0.42** | **3** | **6.79** |  |
| **Mean except Diptera+Lepidoptera** | **5.11** | **3** | **6.89** |  |
